# Investigation of Isocitrate Dehydrogenase 1 and 2 Mutations in Acute Leukemia Patients in Saudi Arabia

**DOI:** 10.3390/genes12121963

**Published:** 2021-12-09

**Authors:** Heba Alkhatabi, Haneen Abdulfattah Bin Saddeq, Luay Alyamani, Thoraia Shinawi, Elrashed B. Yasin, Raed Alserihi, Raed Felimban, Hossam H. Tayeb, Rawan Mimani, Zainab Alalla, Muhammad Abu-Elmagd, Adel Abuzenadah

**Affiliations:** 1 Department of Medical Laboratory Technology, Faculty of Applied Medical Sciences, King Abdulaziz University, Jeddah 80200, Saudi Arabia; halkhattabi@kau.edu.sa (H.A.); tshinawi@kau.edu.sa (T.S.); aaalserihi@kau.edu.sa (R.A.); faraed@kau.edu.sa (R.F.); hhtayeb@kau.edu.sa (H.H.T.); aabuzenadah@kau.edu.sa (A.A.); 2 Center of Excellence in Genomic Medicine Research (CEGMR), King Abdulaziz University, Jeddah 80200, Saudi Arabia; rmaimani111@hotmail.com (R.M.); zainabalalla@yahoo.com (Z.A.); mabuelmagd@kau.edu.sa (M.A.-E.); 3 King Abdullah Medical City, Makkah 24246, Saudi Arabia; Binseddeeq.h@kamc.med.sa; 4 Basic Medical Sciences Department, College of Medicine, King Saud Bin Abdelaziz University for Health Science, Jeddah 80200, Saudi Arabia; loai.a@outlook.com; 5 Department of Medical Laboratory Technology, Faculty of Applied Medical Sciences, King Abdulaziz University, Rabigh 25732, Saudi Arabia; 6 Center of Innovation in Personalized Medicine (CIPM), King Abdulaziz University, Jeddah 80200, Saudi Arabia; 7 King Fahad Medical Research Center (KFMRC), King Abdulaziz University, Jeddah 80200, Saudi Arabia

**Keywords:** IDH1, IDH2, AML, ALL

## Abstract

Different forms of human cancer show mutations for isocitrate dehydrogenases 1 and 2 (IDH1/2). Mutation of these genes can cause aberrant methylation of the genome CpG islands (CGIs), which leads to an increase of suppressed oncogenes transcription or repression of active tumor suppressor gene transcription. This study aimed to identify the prevalence of IDH1/2 mutations in acute leukemia patients. The study cohort included 43 AML patients and 30 childhood ALL patients, from whom DNA bone marrow samples were taken. The alteration hotspots in codons IDH1 (R132) and IDH2 (R172 and R140) were examined via direct sequencing. Mutations in IDH1 were detected in 7 out of 43 (16.2%) AML patients; 5 of them occurred at codon R132. The other two mutations included a single-nucleotide polymorphism, which affected codon G105 in one patient. However, no mutation was detected in the IDH2 in any of the patients. Moreover, no mutations were detected in either IDH1 or IDH2 in ALL patients. The dominance of IDH1 mutations in AML, which was 16%, emphasizes the existence of the mutation in our population. On the other hand, IDH2 mutation was observed to be less frequent in both illnesses. Due to the limitation of using a small sample size, larger cohort screening is recommended to determine their usefulness as prognostic indicators.

## 1. Introduction

Isocitrate dehydrogenase (IDH) 1 and 2 are key Krebs cycle enzymes that are nicotinamide adenine dinucleotide phosphate (NADP+)-dependent, catalyzing the oxidative decarboxylation of isocitrate to α-ketoglutarate (α-KG) [1]. There are three types of IDH, named 1 to 3, that are distinct. IDH1 and IDH2 are found on chromosomes 2q34 and 15q26, correspondingly, while IDH3 is located on chromosomes 15q25 [2]. Mutations in IDH types 1 and 2 were first discovered in 2008 in brain tumor—glioma [3].

IDH1 and IDH2 mutations show several similar clinical properties. Both mostly occur in class two and three gliomas, but not in the lower gliomas [4]. IDH1 and IDH2 mutations are located in cytogenetically normal AML but rarely in other subtypes. The mutations arise at an early phase of tumorigenesis, aligning with the conception that IDH1and IDH2 mutations can affect the determination of cell fate and successive differentiation. In AML and glioma, these mutations and others are linked to better prognosis [4,5]. Both IDH1 and IDH2 normally catalyze the oxidative decarboxylation of isocitrate to α-ketoglutarate; however, the hotspot mutations at R132 in IDH1 and R140 or R172 in IDH2 lead to neomorphic enzymatic activity that, in turn, results in overproduction of D-2-hydroxyglutarate. This oncometabolite has broad effects on cellular biology, including altered metabolism, aberrant DNA and histone methylation, chromatin restructuring, and blocking of the normal differentiation patterns [6].

Mutations of IDH1/2 are heterozygous, comprising an oncogenic improvement in function. The modifications involve variations in missense, leading to the substitution of arginine deposits in exon 4 within the *IDH1/2* genes [7]. In addition, a germline-synonymous single-nucleotide polymorphism (rs11554137) exists in the *IDH1* gene, codon 105 [7]. It appears that the location of the mutation influences IDH activity more than the substituted amino acids do and provides a benefit to the malignant cells that have IDH mutations. In IDH1, replacements at R132 range from cytosine to glycine, while in IDH2, substitutes at R172 include lysine, methionine, tryptophan, and glycine [7].

In myeloid cancers, IDH1/2 mutation has been identified as an induction event [8]. However, IDH1 mutations are mainly involved in early occurrences of AML. Botton et al. (2016) reported that IDH1 modifications are found in about 10% of AML patients and are connected to worse outcomes in patients undergoing thorough chemotherapy. Likewise, IDH2 mutations in de novo AML patients range from 8% to 19% [8]. Thus, IDH2 alteration is more frequent. The prognostic impacts of these mutations vary widely according to the mutational continuum. IDH1/2 alterations occur mainly in normal cytogenetic AML, resulting in the overgeneration of D-2-HG. Additionally, they are naturally heterozygous and mutually exclusive [9]. IDH1 mutation frequently occurs along with other mutations, while IDH2 is detected alone. Most AML conditions with IDH1/2 mutation are grouped morphologically as AML having or not having myeloid maturation, which is represented as FAB (French–American–British) classification M1 or M2, respectively [9]. Moreover, they have morphologic proof of dysplasia.

There is limited knowledge about the incidence of IDH1 and IDH2 mutations in acute lymphoid leukemia (ALL) patients and the prevalence of these mutations in acute leukemia patients in Saudi Arabia. The Saudi Health Council has reported that childhood cancers account for 5.9% of all cancers among Saudis and are considered more common than adult ALL. In 2014 leading cancer type among children in Saudi Arabia was leukemia (34.6%) [10]. Since the initial discovery of mutations in the IDH1 by whole-genome sequencing in a large subset of human gliomas [11] and AML patients [12], there has been a focus on understanding the consequences of mutations in *IDH* genes and their roles in tumor progression. The data revealed the prevalence of DNA methylation-associated gene mutations, and their influence on the Saudi population, is still uncertain. Screening the mutation status of IDH1 and IDH2 in samples from ALL and AML patients will help to understand prognostic significance and prevalence better. In addition, identifying the association between the mutations of these genes in relation to patients’ clinical features and age will provide a better understanding, which will subsequently help when exploring the utilization of IDH1 and 2 as therapeutic targets.

## 2. Materials and Methods

### 2.1. Patients Data

Seventy-three leukemia samples were collected from King Abdullah Medical City (KAMC) and the Center of Excellence in Genomic Medicine Research (CEGMR). Sample data of 43 (25 males and 18 females) newly diagnosed AML patients with an age range from 18 to 76 years were included in this study. In addition, 30 samples (14 males and 16 females) from childhood ALL patients aged between 1 and 14 years were also collected. Patient samples were collected under ethical approval by KAMC IRB registered at the National BioMedical Ethics Committee, King Abdulaziz City for Science and Technology (KACST) (Registration No. H-02-K-001). Informed consent was obtained from all patients. All patient information and clinical data included in this study are listed in Table 1 and Table 2.

### 2.2. Cytogenetic and Molecular Data

The cytogenetic and molecular results were obtained from both CEGMR and KAMC. The investigated molecular markers for AML patients included nucleophosmin (NPM1), fms-like tyrosine kinase (FLT3), BCR/ABL (breakpoint cluster region gene/Abelson proto-oncogene), and promyelocytic leukemia/retinoic acid receptor α (PML/RARA). Molecular results are illustrated in Table 1. For ALL patients, there was no record for molecular tests.

### 2.3. DNA Extraction and PCR Amplification

DNA extraction was performed according to the manufacturer’s instructions for the QIAamp DNA extraction (Qiagen, Germantown, MD, USA) mini kit (250). Touchdown PCR was performed starting with a denaturation step at 95 °C. Annealing was performed at 45–65 °C for 20 to 40 s. Hot Start Taq DNA polymerase (1000 µ) from Qiagen was used to add a nucleotide dNTP set, PCR grade (4 × 250) from Qiagen, to the 3′ end of the primer annealed to the template DNA. Primers’ sequence was as follows: IDH1 (PCR product: 132 bp)—ex4: F: 5′-GTGGAAAAGTCCCAATGG-3′, R: 5′-ACAAGAGGATGGCTAGG-3’; IDH2 (PCR product: 200 bp)—ex4: F: 5′-GTGGAAAAGTCCCAATGG-3′, R: 5′-ACAAGAGGATGGCTAGG-3′.

### 2.4. DNA Sequencing

After genomic DNA amplification by PCR, the product was sequenced to verify its specificity using cycle sequencing. The sequence reaction was performed as follow: 1 μL of the purified PCR product, 2.5 μmol/L of each PCR primer, 2 μL of ABI PRISM terminator cycle sequencing kit version 3.1 (Applied Biosystems, Waltham, MA, USA), and 2 μL 5× sequence buffer to reach a final volume of 10 μL. The sequencing program started from 96 °C for 1 min and was followed by 25 cycles of the following PCR program: denaturation at 96 °C for 10 s, annealing at 50 °C for 5 s, and extension at 60 °C for 4 min. PCR amplicons were sequenced using ABI Prism BigDye kit (PE Applied Biosystems, Foster City, CA, USA) on an ABI3500 system (PE Applied Biosystems, Foster City, CA, USA) by following the manufacture’s protocol. The mutational hotspots in codon 132 of IDH1 on exon 4 were examined through Sanger sequencing. Mutations on IDH1 (IDH1R132, IDH1R100, and IDH1 R109) were screened in all patients.

## 3. Results

### 3.1. IDH1 and IDH2 Mutations Analysis

**Figure 1:**
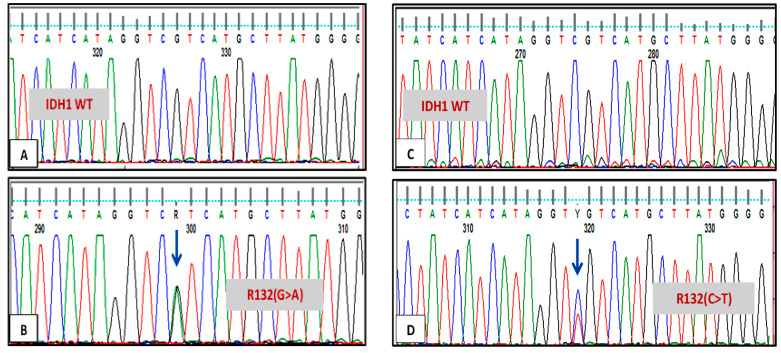
Sequencing result of IDH1 in two different patients. (**A**,**C**) Patient with normal IDH1 sequence at codon R132; (**B**) patient with IDH1 mutation at codon R132 (G>A); (**D**) patient with IDH1 mutation at codon R132 (C>T).

Sequencing 43 AML patients showed that 7 out of 43 patients (16.2%) had IDH1 mutation; 4 of them had a heterozygous missense variant, rs121913500 single-nucleotide polymorphism (SNP), at codon R132. Mutations in IDH1 are summarized in Table 3, and the sequencing result is shown in Figure 1.

**Figure 2:**
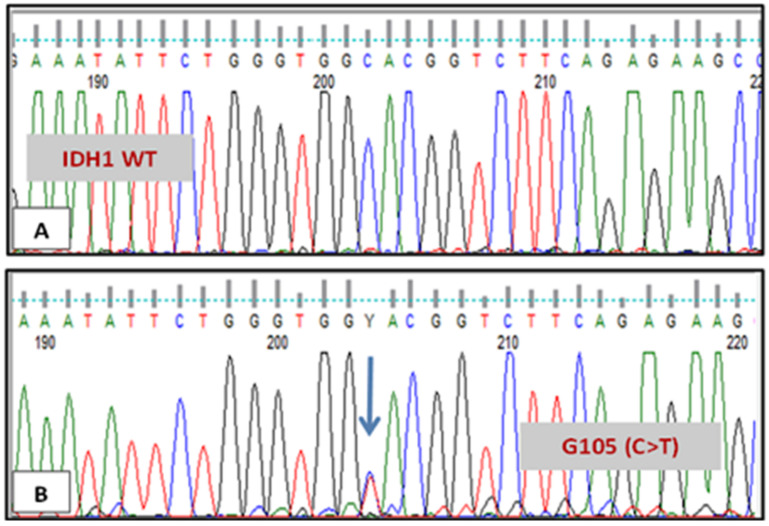
Sequencing result of IDH1 in two different patients. (**A**) Patient with normal IDH1 sequence at codon G105. (**B**) Patient with IDH1 mutation at codon G105 (C>T).

**Figure 3:**
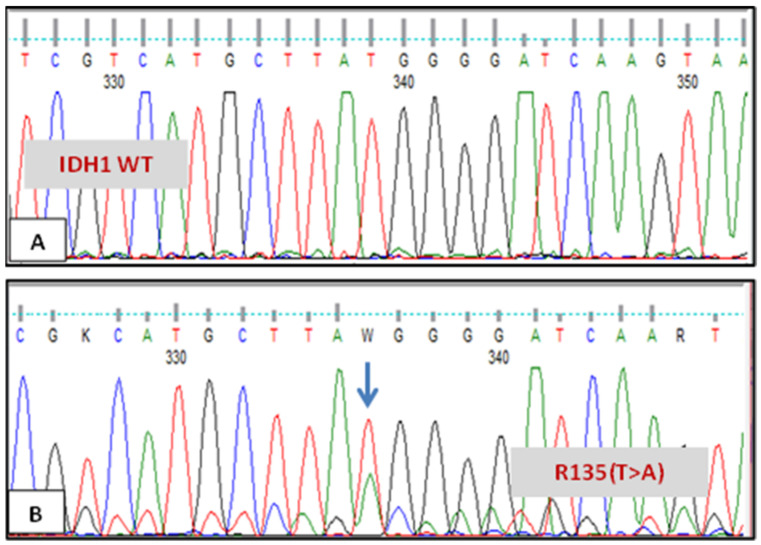
Sequencing chromatogram of IDH1 in two different patients. (**A**) Patient with normal IDH1 sequence at codon R135. (**B**) Patient with IDH1 mutation at codon R135 (T>A).

A synonymous mutation at position G105 of exon 4 replaced a common (GGC) codon with a rare (GGT) codon encoding glycine in patient no. 4. The SNP G105 rs11554137 C>T is shown in Figure 2 compared to the normal sequence. In addition, another synonymous mutation was found (rs767243751), and it was only seen in patient no. 6 at codon R135. This is illustrated in Figure 3. Patient no. 7 had double mutations in *IDH1* gene at codons R132 and G105.

IDH2 (IDH2R172, IDH2 R149, and IDH2140) was screened, and no mutations were detected in AML patients.

However, in ALL samples, there were no mutations detected in both *IDH1* and *IDH2* genes.

### 3.2. Correlation of IDH1 and IDH2 with the Cytogenetic Aberrations

The cytogenetic results showed normal karyotype in 24 out of 43 (56%) AML patients. However, 16 patients were presented with abnormal karyotypes. These included t(8;21) in nine patients and t(15;17) in one patient, inversion 16 in one patient, three patients with different types of trisomies, and two patients with a complex karyotype. For ALL patients, 50% had a normal karyotype, one (3%) patient had t(9;22), and three patients had complex karyotype, which included trisomy t(9;11), t(1;3), del(3), and ins(11). The remaining patients had no records for the cytogenetic result.

Among patients with IDH1 R132 mutation, all patients except two showed a normal karyotype. One of the two cases with cytogenetic abnormality showed trisomy 4, 8, and 21, and the other had t(8;21) and 2q deletion. On the other hand, IDH1 mutation silent SNP G105 was associated with a complex karyotype that included translocation (9;11) and trisomy 13, 21, and 22. In addition, for R135, the patient had a normal karyotype (Table 3).

### 3.3. Correlation of IDH1/2 with Molecular Results

NPM1 mutation was detected in two patients; one had IDH1 mutation at codon R132 (c.394C>T, p.Arg131Cys), while the other had a mutation in IDH2 c.612 + 45G>A, both patients had a normal cytogenetic result.

### 3.4. IDH1 and IDH2 Mutations Association with the Treatment Response

All investigated patients were treated according to their disease classification and phenotype. In AML patients, 28 (65%) showed an improved response and achieved complete remission, whereas 15 patients (35%) went through relapses. In ALL patients, 19 patients (63%) were in remission, and 11 patients (37%) were either at diagnosis or in relapse stage.

For patients with IDH1 mutation, four out of seven of them showed improved treatment response, and the remaining three, which included codon R132 c.395G>A, pArg13His, codon G105, c.315C>T, pGly105Gly and codon R132, c.394C>T, p.Arg131Cys + codon G105, c.315C>T, pGly105Gly, were on relapse.

## 4. Discussion

The *IDH1* gene localizes in the cytoplasm of the cell. IDH1 and 2 help the cells to produce energy through IDH1 enzymes, which catalyze the conversion of isocitrate to α-ketoglutarate, utilizing NADPH to protect the cells from the toxic effect of the reactive oxygen [1]. In addition to its role in metabolism, α-ketoglutarate is an important substrate for the cytoplasmic and nuclear α-ketoglutarate-dependent enzyme to carry out the normal function. In the nuclease, this enzyme helps in regulating the DNA and histone methylation pattern, which, in turn, switches the expression of the genes on and off, including genes critical for cellular differentiation [1]. In IDH mutant cancer, the IDH mutant enzyme acquires a new function (gain of function activity) that disrupts many cellular functions. The mutated copy of the enzyme converts α-ketoglutarate to hydroxyglutarate, which alters the histone methylation, leading to DNA damage due to impaired DNA repair. This hypermethylation effect alters the transcription of key genes involved in cellular differentiation, accumulates immature cells, and contributes to oncogenesis [1].

The data obtained from this integrated exploration of mutations and alterations have presented a different interpretation of AML and ALL. Out of 43 AML patients, 7 had IDH1 mutations while 5 showed a heterozygous missense alteration at codon R132, and one of them had an additional mutation at codon G105. Moreover, other silent SNPs at codon G105 and R135 were identified in different samples. There was no mutation detected on IDH2 in all investigated samples. For ALL patients, there were no mutations detected on either IDH1 or IDH2 in all patients.

The prevalence of IDH1 and IDH2 alterations in cytogenetically normal AML patients is higher relative to cytogenetically abnormal AML patients. This observation implies that AML is linked with a cytogenetically normal karyotype. In testing the correlation of IDH1 with cytogenetic outcomes, most participants with IDH1 mutation at R132 had a normal karyotype. It was in remission, except for one patient who had an additional mutation in IDH1 and failed to go into remission. There was only one patient with IDH1 mutation at R132, and they had cytogenetic defects that could have been the cause of going into relapse. Furthermore, another patient with an abnormal karyotype (t (8;21)) entered into complete remission. These findings show that IDH mutations provide a favorable prognosis in the presence or absence of a normal karyotype. This trend indicates that the impact of IDH1 mutations on tumorigenesis may be related to the determination of cell fate at an explicit stage of cell differentiation. Knowing the gene function, mutations in the gene influence the production of D-2-hydroxyglutarate, which in the AML would likely block the maturation of the cells, resulting thus in overproduction of immature cells and tumor formation. The mutations strike at early phases of tumorigenesis. Likewise, IDH1 mutations impair the identification of cell fate and the successive distinction. Yang et al. (2012) reported that in AML and glioma, the alterations alone or together with other cytogenetic deformities could lead to a better prognosis [1].

The existence of silent SNP at codon G105 in *IDH1* in two patients was associated with a poor diagnosis, and the patients who had this SNP failed to go into remission. This trend might be due to the complicated cytogenetic malformation that one patient had. The other patient had a normal cytogenetic result and had an additional missense variant at codon R132 in the *IDH1* gene. Both patients showing evidence normally associated with a good, not poor, response. However, the condition through which a silent SNP can change the function of a gene, such as the limitation of mRNA stability or appearance, is presently under research. The substitution of a codon with another one that a synonymous SNP rarely uses may slow down the protein translation rate, leading to transformed protein folding and, eventually, reduced function of proteins. Despite the ongoing study in the biology of matching SNPs in AML, Phoenix et al. (2011) suggested that several SNPs in AML-related genes are not medically inactive [13]. This observation implies that identical SNPs are among the molecular alterations that need to be examined in AML for disease categorization and diagnosis. Another notable finding in the current study is the presence of silent SNP in only one sample at position R135. That patient had normal cytogenetic results, no molecular changes, and was going through remission.

In the molecular analysis, for AML patients, only one had the *NPM1* gene mutation, which shows distinctive biological and medical features.

IDH1 and IDH2 alterations are mainly linked and co-occur with *NPM1* transformation. Patients with the *NPM1* mutation with no FLT3 internal tandem duplication (ITD) or low FLT3-ITD levels had an excellent response to chemotherapy and a favorable prognosis [14]. However, the prognostic significance of these mutations varies significantly, following the mutational range [8]. Likewise, Patel et al. (2011) reported that other alterations frequently accompany IDH1, while IDH2 is identified primarily alone [15]. In the present study, most patients with IDH1 mutation had no molecular changes, except for one patient with *NPM1* who showed a normal karyotype, indicating the patient’s survival rate. However, IDH1 alterations may suggest a higher possibility of relapse and low chances of survival in cytogenetically healthy patients with altered *NPM1*.

In addition, IDH2 mutations at various codons, including IDH2-R172, IDH2-R149, and IDH2-R140, have been tested in the current study but were not detected in any of the samples. While it can be argued that IDH2 mutations rarely exist in AML mutations, previous studies suggested otherwise. Ward et al. (2010) reported that IDH2 was altered in AML through sequencing of the *IDH2* gene in a collection of specified AML samples [16]. In their study, several IDH-R172 alterations were found. Initially, R172K was identified in glioma patients. Moreover, it has been indicated that cancer-related IDH1-R132 transformations show a loss of role for the utilization of isocitrate as substrate and a gain of function in the reduction of α-KG to D-2-HG. Following this finding, Ward et al. (2010) studied the presence of a similar neomorphic process in IDH2-R172K mutation observed in AML and gliomas [16].

For ALL, multiple studies showed that changes in IDH1and IDH2 were associated with poor results on lymphoid malignancies [17,18]. Andersson et al. found IDH1 and IDH2 mutations in 1 out of 288 pediatric ALL patients [19], and this supports our finding of the low incidence of IDH1 and IDH2 in ALL patients. In their study, Simonin et al., 2021, also found a rare incidence of IDH1/2 in ALL patients. Even though they were using a sensitive method, targeted next-generation sequencing, they found a mutation in 4% of their investigated samples, which included 1085 patients. Furthermore, IDH2 was more prominent than IDH1, which is common in AML and older ALL patients [20].

According to our study using a small sample size, no mutations were detected. The lack of these genes in these samples further indicates that changes in these genes may not play an active part in the pathogenesis of ALL in childhood patients. These genes’ mutations were selected because they play an important role in proper prognosis and survival among patients suffering from other diseases (acute myeloblastic leukemia, myelodysplastic syndromes, gliomas, and others) [19]. The clinical impact of the genetic variations needs to be investigated to enhance the efficiency of treating childhood leukemia. Previous studies conducted in different regions or countries have led to different results. Therefore, since Saudi Arabia is a multiethnic society, we cannot generalize these findings; future studies should consider other areas of Saudi Arabia to clarify the whole picture of these mutations in the Saudi Arabia population.

Research in recent decades has indicated an enormous improvement in discovering new molecular mutations and designing targeted therapy, which is of high clinical significance in hematological malignancies [21,22]. A recent study by Kaminska, Czapski, Guzik, Król, and Gielniewski (2019) showed that IDH1-R132 consists of an immunogenic epitope, which can be used in the creation of mutant protein-definite vaccines. In assessing the effectiveness of the immunization, humanized mice containing IDH1-R132 lumps were used, which resulted in the production of antitumor immune reaction and limitation of progression of IDH1-R132 tumors. Thus, IDH1-R132 is a suitable target for therapy [23]. Despite the limitation of our sample size, this study has critically highlighted the prevalence of IDH1 and IDH2 in leukemia patients in the Saudi population. This knowledge may provide a starting point for developing new, useful markers for medical testing and a safer individualized medication for patient treatment. Further investigations with a larger sample size associated with screening all exons of *IDH1* and *IDH2* genes rather than screening hotspots using highly sensitive techniques could give a generalized conclusion, further risk stratification, and effective patient management.

## 5. Conclusions

The dominance of IDH1 mutations in AML, which was 16%, emphasizes the existence of the mutation in our population. Additionally, IDH1-R132 was prevalent, with a favorable prognosis and normal cytogenetic result, in AML patients. However, there were no mutations on *IDH1* in ALL patients. Furthermore, *IDH2* mutation was observed to be less frequent in both illnesses. Therefore, despite the limitation of using a small sample size, our study has critically compared *IDH1/2* alterations in leukemia patients in one region. A larger cohort screening is recommended to determine their usefulness as prognostic indicators.

## Figures and Tables

**Table 1:** Clinical information of AML patients.

Age, years, median (range)	(18–76) 34
Gender (female/male)	18/25
**Cytogenetic, n (%)**	
normal	24 (56%)
single abnormality	16 (37%)
complex	2 (5%)
unknown	1 (2%)
**Molecular Changes (%)**	
FLT3/ITD	2 (5%)
FLT3/TKD	2 (5%)
NPM1	5 (12%)
BCR/ABL	2 (5%)
PML/RARA	2 (5%)
None	31 (72%)
**Outcome Following Treatment**	
In remission	28 (65%)
Relapse	15 (35%)

**Table 2:** Clinical information of ALL patients.

Age, years, median (range)	(1–14) 8
Gender (female/male)	16/14
**Cytogenetic, n (%)**	
normal	15 (50%)
single abnormality	1 (3%)
complex	3 (10%)
unknown	11 (37%)
**Outcome Following Treatment**	
In remission	19 (63%)
Relapse	11 (37%)

**Table 3:** IDH1/2 mutations in AML patients.

Patient No.	Age/Sex	Diagnosis	Karyotype	Type of IDH Mutation	Presence of Other Mutations	Treatment Response
1	31/F	AML	trisomy 4,8 and 21	IDH1 codon R132 c.395G>A, pArg13His	none	Relapse
2	17/F	AML	cytogenetically normal	IDH1 codon R132, c.394C>T, p.Arg131Cys	NPM1	In remission
3	21/M	AML	t(8:21)(q22:q22), -2q deletion	IDH1 codon R132 c.395G>A, pArg13His	none	In remission
4	29/F	AML	Complex karyotype: translocation (9;11), trisomy 13,12 and 22	IDH1 codon G105, c.315C>T, pGly105Gly	none	Relapse
5	18/F	AML	cytogenetically normal	IDH1 codon R132, c.394C>T, p.Arg131Cys	none	In remission
6	36/F	AML	cytogenetically normal	IDH1 codon R135 c. T>A,	none	In remission
7	22/M	AML	cytogenetically normal	IDH1 codon R132, c.394C>T, p.Arg131Cys + codon G105, c.315C>T, pGly105Gly	none	Relapse

## Data Availability

The data presented in this study are available on request from the corresponding author.
